# Supplementary material for: ProteinDJ: A high‐performance and modular protein design pipeline
Source: Protein Sci. 2026 Jan 21;35(2):e70464. doi: 10.1002/pro.70464 (PMC12820799; doi:10.1002/pro.70464)
Supplement: Supplementary file 1 — TABLE S1. The containers and software dependencies of ProteinDJ. TABLE S2. The filtering parameters available in ProteinDJ. TABLE S3. ProteinDJ outputs a wide array of additional metrics to assist researchers to evaluate potential designs. Some metrics are calculated by software in the pipeline (+), but there are additional metrics calculated using PyRosetta (Sappington et al., 2024) and BioPython (Vazquez Torres et al., 2025). TABLE S4. Parameters for RFdiffusion used for different modes. Parameters are ignored if they are not relevant to the design type or cause conflicts. TABLE S5. Scaffold templates for fold conditioning. TABLE S6. Benchmarking target structures. Structures were prepared including the same residues as used for RFdiffusion benchmarking in Watson et al. (Watson et al., 2023). The contigs and hotspots provided to RFdiffusion are indicated below. FIGURE S1. Example execution of ProteinDJ pipeline for de novo binder design. (a) Header with key parameters and output directory. (b) Description of contigs provided and how these will be used in diffusion. (c) Standard Nextflow process tracker that updates as each task completes. (d) A summary of the run, highlighting success rates at each stage. FIGURE S2. ProteinDJ multi‐GPU efficiency and scaling. (a) Each data point represents the total wall‐clock time excluding queue time for a complete ProteinDJ pipeline execution (4000 designs) using the specified number of NVIDIA A30 GPUs. (b) Parallel efficiency of ProteinDJ calculated relative to single GPU execution. Each data point represents the parallel efficiency percentage calculated from wall‐clock execution times. [file PRO-35-e70464-s002.docx]

## Supplementary Material

# ProteinDJ: a high-performance and modular protein design pipeline

Dylan Silke^1,2^, Julie Iskander^1,2^, Junqi Pan^1,2^, Andrew P. Thompson^1,2^, Anthony T. Papenfuss^1,2^, Isabelle S. Lucet^1,2*^, Joshua M. Hardy^1,2*^

1. The Walter and Eliza Hall Institute of Medical Research, Parkville, VIC, Australia.
2. Department of Medical Biology, University of Melbourne, Parkville, VIC, Australia.

*Co-corresponding authors: Isabelle S. Lucet ([lucet.i@wehi.edu.au](mailto:lucet.i@wehi.edu.au)), Joshua M. Hardy ([hardy.j@wehi.edu.au](mailto:hardy.j@wehi.edu.au))

Supplementary Table 1 - The containers and software dependencies of ProteinDJ.

| **Container** | **Description** | **Repository** |
| --- | --- | --- |
| af2 | A modified version of AlphaFold2 (Initial Guess) as implemented in dl_binder_design. Used for structure prediction and design validation. | https://github.com/PapenfussLab/dl_binder_design.git |
| bindcraft | Contains the BindCraft binder design pipeline that includes AlphaFold2, ColabFold, ProteinMPNN and PyRosetta. | https://github.com/martinpacesa/BindCraft |
| boltz2 | Structure prediction design validation and filtering using Boltz-2 (inference only). | https://github.com/jwohlwend/boltz.git |
| dl_binder_design | Environment for ProteinMPNN FastRelax used for sequence design. Includes ‘vanilla’ and ‘soluble’ checkpoint models and implementation of Rosetta FastRelax Protocol | https://github.com/PapenfussLab/dl_binder_design.git |
| fampnn | Environment for Full-Atom MPNN, a sequence design tool. | https://github.com/PapenfussLab/fampnn.git |
| pyrosetta_tools | Python interface to Rosetta molecular modeling suite (PyRosetta) bundled with standard data analysis libraries (BioPython, NumPy, Pandas, Matplotlib) for processing pipeline results. | N/A |
| python_tools | Lightweight container with standard python data analysis libraries (BioPython, NumPy, Pandas, Matplotlib) for processing pipeline results. | N/A |
| rfdiffusion | Contains RFdiffusion, a fold design software integral to the pipeline | https://github.com/PapenfussLab/RFdiffusion |

Supplementary Table 2 – The filtering parameters available in ProteinDJ.

| **Parameter** | **Description** |
| --- | --- |
| **RFdiffusion** |  |
| fold_min_helices | Minimum number of alpha-helices required. |
| fold_max_helices | Maximum number of alpha-helices allowed. |
| fold_min_strands | Minimum number of beta-strands required. |
| fold_max_strands | Maximum number of beta-strands allowed. |
| fold_min_ss | Minimum number of secondary structure elements (α-helices + β-strands). |
| fold_max_ss | Maximum number of secondary structure elements (α-helices + β-strands). |
| fold_min_rog | Minimum radius of gyration (Å). |
| fold_max_rog | Maximum radius of gyration (Å). |
| **ProteinMPNN** |  |
| mpnn_max_score | Maximum MPNN score (negative log likelihood). |
| **FAMPNN** |  |
| fampnn_max_psce | Max predicted-side chain error (PSCE) score for designed side-chains. |
| **AlphaFold2 Initial Guess** |  |
| af2_max_pae_interaction | Max predicted aligned error for interactions |
| af2_max_pae_overall | Max predicted aligned error for all chains |
| af2_max_pae_binder | Max predicted aligned error for binder |
| af2_max_pae_target | Max predicted aligned error for target |
| af2_min_plddt_overall | Max C-alpha RMSD of binder when binder chains are aligned |
| af2_min_plddt_binder | Max C-alpha RMSD of binder when target chains are aligned |
| af2_min_plddt_target | Max C-alpha RMSD of target when target chains are aligned |
| af2_max_rmsd_overall | Max C-alpha RMSD when all chains are aligned |
| af2_max_rmsd_binder_bndaln | Max C-alpha RMSD of binder when binder chains are aligned |
| af2_max_rmsd_binder_tgtaln | Max C-alpha RMSD of binder when target chains are aligned |
| af2_max_rmsd_target | Max C-alpha RMSD of target when target chains are aligned |
| **Boltz-2** |  |
| boltz_max_rmsd_overall | Max C-alpha RMSD between all chains of Boltz-2 prediction and RFD design |
| boltz_max_rmsd_binder | Max C-alpha RMSD between binder chains of Boltz-2 prediction and RFD design. |
| boltz_max_rmsd_target | Max C-alpha RMSD between target chains of Boltz-2 prediction and RFD design. |
| boltz_min_conf_score | Minimum confidence score of the prediction |
| boltz_min_ptm | Minimum predicted template modelling (pTM) score of the prediction |
| boltz_min_ptm_interface | Minimum pTM-score of the prediction interface |
| boltz_min_ptm_binder | Minimum pTM-score of the binder chain |
| boltz_min_ptm_target | Minimum pTM-score of the target chain |
| boltz_min_plddt | Minimum pLDDT score of the prediction |
| boltz_min_plddt_interface | Minimum pLDDT score of the prediction interface |
| boltz_max_pde | Maximum predicted distance error of the prediction |
| boltz_max_pde_interface | Maximum predicted distance error of the prediction interface |
| boltz_min_ipSAE_min | Minimum value allowed for the minimum interaction prediction Score from Aligned Errors (ipSAE) of target and binder chains |
| boltz_min_LIS | Minimum Local Interaction Score (LIS) |
| boltz_min_pDockQ2_min | Minimum value allowed for the minimum predicted DockQ Score v2 of target and binder chains. |
| boltz_max_pae_interaction | Maximum predicted aligned error at interaction interfaces |
| **PyRosetta/BioPython** |  |
| pr_min_helices | Minimum number of alpha-helices in predicted structure |
| pr_max_helices | Maximum number of alpha-helices in predicted structure |
| pr_min_strands | Minimum number of beta-strands in predicted structure |
| pr_max_strands | Maximum number of beta-strands in predicted structure |
| pr_min_total_ss | Minimum total secondary structure elements (α-helices + β-strands) in predicted structure |
| pr_max_total_ss | Maximum total secondary structure elements (α-helices + β-strands) in predicted structure |
| pr_min_rog | Minimum radius of gyration (Å) of predicted structure |
| pr_max_rog | Maximum radius of gyration (Å) of predicted structure |
| pr_min_intface_bsa | Minimum buried surface area (Å²) at the binding interface |
| pr_min_intface_shpcomp | Minimum shape complementarity of interface (0-1 scale; 1 is optimal) |
| pr_min_intface_hbonds | Minimum number of hydrogen bonds at the interface |
| pr_max_intface_unsat_hbonds | Maximum number of buried, unsatisfied hydrogen bonds at the interface |
| pr_max_intface_deltag | Maximum solvation free energy gain at interface (Rosetta Energy Units; lower is better) |
| pr_max_intface_deltagtobsa | Maximum ratio of delta-G to buried surface area |
| pr_min_intface_packstat | Minimum packing quality of the interface (0-1 scale; higher is better) |
| pr_max_tem | Maximum total energy metric score (Rosetta Energy Units; lower indicates more stable designs) |
| pr_max_surfhphobics | Maximum percentage of hydrophobic residues exposed on the surface |
| pr_max_sap | Maximum mean residue Spatial Aggregation Propensity for monomer/binder (solubility prediction; lower is better) |
| pr_max_sap_complex | Maximum mean residue Spatial Aggregation Propensity for binder in complex (solubility prediction; lower is better) |
| seq_min_ext_coef | Minimum extinction coefficient at 280nm (M⁻¹cm⁻¹) |
| seq_max_ext_coef | Maximum extinction coefficient at 280nm (M⁻¹cm⁻¹) |
| seq_min_pi | Minimum isoelectric point (pI) of the sequence |
| seq_max_pi | Maximum isoelectric point (pI) of the sequence |

Supplementary Table 3 – ProteinDJ outputs a wide array of additional metrics to assist researchers to evaluate potential designs. Some metrics are calculated by software in the pipeline (^+^), but there are additional metrics calculated using PyRosetta^18^ and BioPython^19^.

| **Metric name** | **Explanation** |
| --- | --- |
| description | Unique filename of structure prediction |
| fold_id | Fold ID for independent execution of RFdiffusion |
| seq_id | Sequence ID for independent execution of ProteinMPNN |
| rfd_sampled_mask^+^ | Contigs used by RFdiffusion to produce this fold (e.g., ['E6-155/0', '100-100']) |
| bc_length | Length of binder selected by BindCraft |
| bc_plddt | Average per-residue confidence score (0-100) for the complex calculated by BindCraft. |
| bc_rmsd_target | The RMSD between the input target structure and the target structure after binder design |
| fold_helices | Number of α-helices in designed fold |
| fold_strands | Number of β-strands in designed fold |
| fold_total_ss | Total secondary structures (helices + strands) in designed fold |
| fold_RoG | Radius of gyration (RoG) for designed fold (Å) |
| rfd_time | Time (seconds) taken by RFdiffusion to produce a fold |
| bc_time | Time (seconds) taken by BindCraft to produce a fold |
| fampnn_avg_psce | Average predicted sidechain error (PSCE) from FAMPNN |
| mpnn_score^+^ | ProteinMPNN's average negative log likelihood |
| af2_pae_interaction^+^ | AF2 PAE for binding interface residue pairs (Å) |
| af2_pae_overall | AF2 PAE for all residue positions (Å) |
| af2_pae_binder^+^ | AF2 predicted aligned error (PAE) for binder residue positions (Å) |
| af2_pae_target^+^ | AF2 PAE for target protein residue positions (Å) |
| af2_plddt_overall^+^ | Average predicted Local Distance Difference Test (pLDDT) score for all residues |
| af2_plddt_binder^+^ | Average pLDDT score for binder residues |
| af2_plddt_target^+^ | Average pLDDT score for target residues |
| af2_rmsd_overall | C-alpha RMSD comparing RFdiffusion design and AF2 prediction (all chains). |
| af2_rmsd_binder_tgtaln^+^ | C-alpha RMSD between RFD binder and AF2 binder when target chain is aligned (Å) |
| af2_rmsd_binder_bndaln^+^ | C-alpha RMSD between RFD binder and AF2 binder when binder chain is aligned (Å) |
| af2_rmsd_target | C-alpha RMSD between RFD target and AF2 target when binder chain is aligned (Å) |
| af2_time^+^ | Time (seconds) for AlphaFold2 prediction |
| boltz_ rmsd_overall | C-alpha RMSD between all chains of Boltz-2 prediction and RFD design (Å) |
| boltz_rmsd_binder | C-alpha RMSD between binder chains of Boltz-2 prediction and RFD design (Å) |
| boltz_rmsd_target | C-alpha RMSD between target chains of Boltz-2 prediction and RFD design (Å) |
| boltz_conf_score^+^ | Confidence score for Boltz-2 prediction |
| boltz_ipSAE_min | Minimum interaction Prediction Score from Aligned Errors (ipSAE) of target and binder chains |
| boltz_LIS | Local Interaction Score (LIS) |
| boltz_pDockQ2_min | Minimum predicted DockQ Score v2 (calculated from PAE) of target and binder chains. |
| boltz_pae_interaction | Predicted aligned error for interaction interfaces. |
| boltz_ptm^+^ | The predicted template modelling (pTM) score for the Boltz-2 prediction |
| boltz_ptm_interface^+^ | Interface pTM-score for Boltz-2 prediction |
| boltz_ptm_binder^+^ | Predicted template modelling score of binder chain |
| boltz_ptm_target^+^ | Predicted template modelling score for target chain |
| boltz_plddt^+^ | pLDDT score for Boltz-2 prediction |
| boltz_plddt_interface^+^ | Interface pLDDT score for Boltz-2 prediction of the complex |
| boltz_pde^+^ | Predicted distance error (PDE) for Boltz-2 prediction |
| boltz_pde_interface^+^ | Interface PDE for Boltz-2 prediction of the complex (Å) |
| pr_helices | Number of α-helices in predicted structure |
| pr_strands | Number of β-strands in predicted structure |
| pr_total_ss | Total secondary structures (helices + strands) in predicted structure |
| pr_RoG | RoG of predicted structure (Å) |
| pr_intface_BSA | Buried surface area (BSA) at binding interface (Å²) |
| pr_intface_shpcomp | Interface shape complementarity (0-1, 1=optimal) |
| pr_intface_hbonds | Number of interface hydrogen bonds |
| pr_intface_unsat_hbonds | Number of buried, unsatisfied hydrogen bonds at the interface. |
| pr_intface_deltaG | Solvation free energy gain (delta G) at interface (kcal/mol) |
| pr_intface_deltaGtoBSA | Ratio of delta-G to buried surface area |
| pr_intface_packstat | Interface packing quality (0-1, higher=better) |
| pr_TEM | Rosetta Total Energy Metric (TEM) score of design (lower=more stable) |
| pr_surfhphobics_% | Percentage of hydrophobic residues on design surface |
| pr_SAP | Mean residue Spatial Aggregation Propensity of monomer/binder (solubility prediction; lower is better). |
| pr_SAP_complex | Mean residue Spatial Aggregation Propensity of binder when complexed with target (solubility prediction; lower is better). |
| seq_ext_coef | Extinction coefficient (M⁻¹cm⁻¹) at 280nm |
| seq_length | Number of amino acids in design |
| seq_MW | Molecular weight (MW) of design (Da) |
| seq_pI | Isoelectric point (pI) of design |
| sequence | Amino acid sequence of design |

+Existing metrics from RFdiffusion, ProteinMPNN-Fast Relax, AlphaFold 2 Initial Guess and Boltz-2

Supplementary Table 4 – Parameters for RFdiffusion used for different modes. Parameters are ignored if they are not relevant to the design type or cause conflicts.

| **Parameter** | **monomer_denovo** | **monomer_foldcond** | **monomer_motifscaff** | **monomer_partialdiff** | **binder_denovo** | **binder_foldcond** | **binder_motifscaff** | **binder_partialdiff** |
| --- | --- | --- | --- | --- | --- | --- | --- | --- |
| **design_length** | **Required** | Ignored | Ignored | Ignored | **Required** | Ignored | Ignored | Ignored |
| **input_pdb** | Ignored | Ignored | **Required** | **Required** | **Required** | **Required** | **Required** | **Required** |
| **hotspot_residues** | Ignored | Ignored | Ignored | Ignored | *Optional* | *Optional* | Ignored | Ignored |
| **rfd_contigs** | *Optional* | Ignored | **Required** | *Optional* | *Optional* | Ignored | **Required** | *Optional* |
| **rfd_scaffold_dir** | Ignored | **Required** | Ignored | Ignored | Ignored | **Required** | Ignored | Ignored |
| **rfd_mask_loops** | Ignored | *Optional* | Ignored | Ignored | Ignored | *Optional* | Ignored | Ignored |
| **rfd_inpaint_seq** | Ignored | Ignored | *Optional* | Ignored | Ignored | Ignored | *Optional* | Ignored |
| **rfd_length** | Ignored | Ignored | *Optional* | Ignored | Ignored | Ignored | *Optional* | Ignored |
| **rfd_partial_diffusion_timesteps** | Ignored | Ignored | Ignored | **Required** | Ignored | Ignored | Ignored | **Required** |
| **rfd_ckpt_override** | *Optional* | *Optional* | *Optional* | *Optional* | *Optional* | *Optional* | *Optional* | *Optional* |
| **rfd_noise_scale** | *Optional* | *Optional* | *Optional* | *Optional* | *Optional* | *Optional* | *Optional* | *Optional* |

Supplementary Table 5 - Scaffold templates for fold conditioning

| **Prefix** | **Description** | **Count** |
| --- | --- | --- |
| HHH | "3-helical bundles" | 17668 |
| HHHH | "4-helical bundles" | 3499 |
| HEEHE | "Mixed alpha-helical (H) and beta-strand (E) scaffold. Artificial." | 208 |
| EHEEHE | "Mixed alpha-helical (H) and beta-strand (E) scaffold based on ferredoxin" | 1555 |

**Supplementary Table 6** – Benchmarking target structures. Structures were prepared including the same residues as used for RFdiffusion benchmarking in Watson et al.^5^. The contigs and hotspots provided to RFdiffusion are indicated below.

| **Protein (Acronym)** | **Biological function** | **PDB ID** | **RFdiffusion contigs** | **RFdiffusion hotspots** |
| --- | --- | --- | --- | --- |
| Influenza A H1 haemagglutinin (HA) | Viral surface protein for host cell attachment and entry. | 5VLI | [A4-53/A79-83/A110-114/A261-325/0 B501-568/B580-670/0 50-100] | B521, B545, B552 |
| Interleukin-7 receptor alpha (IL-7Rα) | Receptor for IL-7, important for lymphocyte development. | 3DI3 | [B17-209/0 50-100] | B58, B80, B139 |
| Insulin Receptor (InsR) | Insulin receptor, regulates glucose uptake and metabolism. | 4ZXB | [E6-155/0 50-100] | E64, E88, E96 |
| Programmed cell death 1 ligand 1 (PD-L1) | Immune checkpoint, protein helps regulate immune responses. | 5O45 | [A17-131/0 50-100] | A56, A115, A123 |
| Tropomyosin receptor kinase A (TrkA) | Receptor for nerve growth factor, promotes neuron survival and growth. | 1WWW | [X282-382/0 50-100] | X294, X296, X333 |


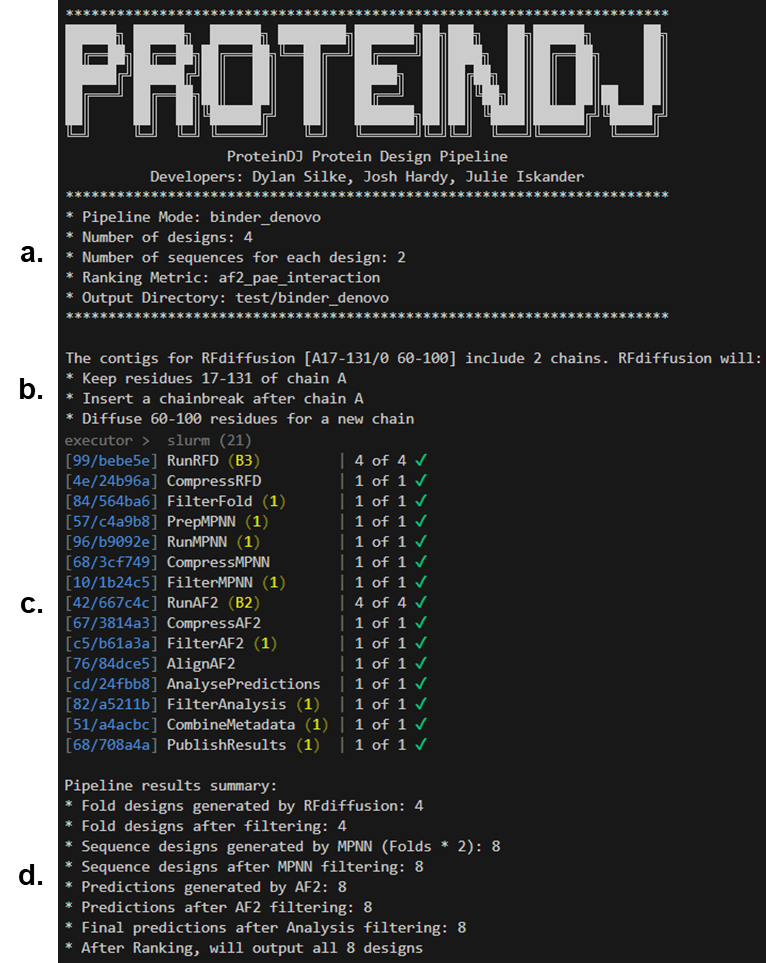


Supplementary Figure 1 – Example execution of ProteinDJ pipeline for *de novo* binder design. a. Header with key parameters and output directory. b. Description of contigs provided and how these will be used in diffusion. c. Standard Nextflow process tracker that updates as each task completes. d. A summary of the run, highlighting success rates at each stage.


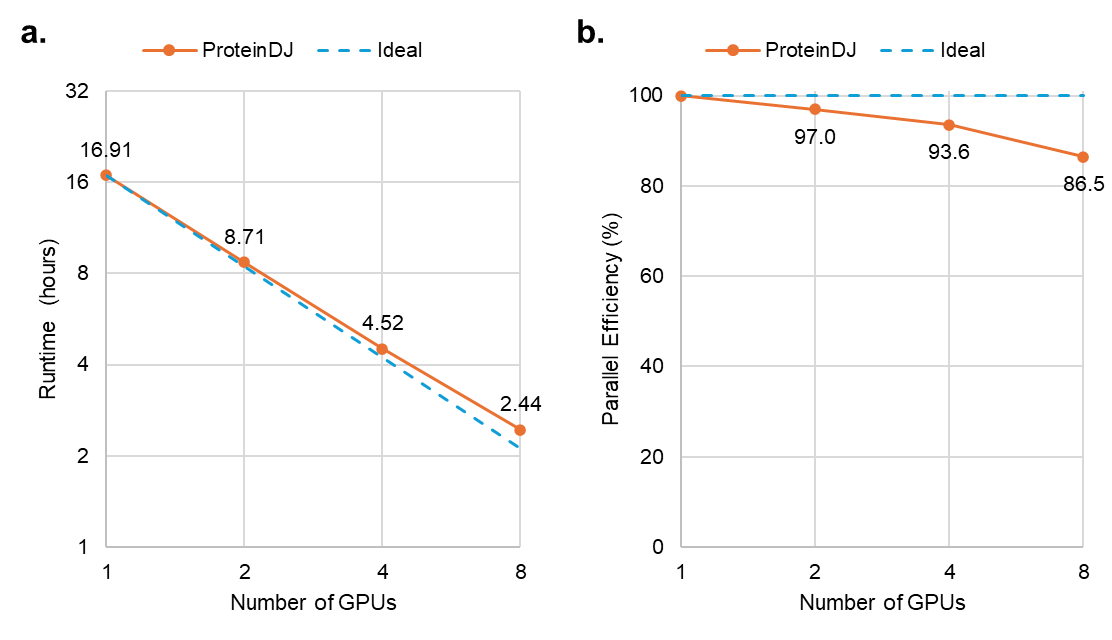


Supplementary Figure 2 – ProteinDJ multi-GPU efficiency and scaling. (a) Each data point represents the total wall-clock time excluding queue time for a complete ProteinDJ pipeline execution (4,000 designs) using the specified number of NVIDIA A30 GPUs. (b) Parallel efficiency of ProteinDJ calculated relative to single GPU execution. Each data point represents the parallel efficiency percentage calculated from wall-clock execution times.

Supplementary File 1 – Compressed archive containing input PDB and Bindsweeper YAML files used for benchmarking and comparison of sequence design methods.
